# Competitor densities, habitat, and weather: effects on interspecific interactions between wild deer species

**DOI:** 10.1111/1749-4877.12470

**Published:** 2020-08-19

**Authors:** Francesco FERRETTI, Niccolò FATTORINI

**Affiliations:** ^1^ Research Unit of Behavioural Ecology Ethology and Wildlife Management Department of Life Sciences University of Siena Siena Italy

**Keywords:** interspecific competition, deer, niche partitioning, rainfall, ungulates, *Dama dama*, *Capreolus capreolus*

## Abstract

There is a growing interest on the potential interplay between weather, habitat, and interspecific competition on population dynamics of wild herbivores. Favorable environmental conditions may buffer the negative effects of competition; conversely, competition may be expected to be stronger under harsh environmental conditions. We investigated relationships between competitor abundance, weather, and habitat cover on density and local distribution of a medium‐sized herbivore, the roe deer *Capreolus capreolus*, as well as its spatial overlap with fallow deer *Dama dama* in a Mediterranean protected area. Over 11 years (2007–2017), roe deer density was not affected by spring–summer rainfall in the previous year and decreased with increasing density of fallow deer in the previous year. Hence, over the considered temporal scale, results supported a major role of competition over weather in influencing population trends of roe deer. At a finer spatial scale, roe deer occupancy was negatively affected by local abundance of fallow deer, especially in “poorer” habitats. We found a slight support for a positive effect of fallow deer density on interspecific spatial overlap. Moreover, fine‐scale spatial overlap between deer species increased with decreasing rainfall in spring–summer. Fallow deer were introduced to our study area in historical times and their role as superior competitors over roe deer has been found also in other study areas. We suggest a potential role of harsh weather conditions during the growing season of vegetation (i.e. scarce rainfall) in triggering the potential for ecological overlap, emphasizing the negative effects of interspecific competition.

## Graphical abstract

We investigated relationships between competitor abundance, weather and habitat on density and distribution of the roe deer Capreolus capreolus, as well as its spatial overlap with fallow deer Dama dama in a Mediterranean protected area. Over 11‐years, roe deer density was not affected by spring‐summer rainfall in the previous year and decreased with increasing density of fallow deer in the previous year. At a finer spatial scale, roe deer occupancy was negatively affected by local abundance of fallow deer, especially in “poorer” habitats; interspecific spatial overlap was influenced both by habitat and rainfall, being negatively related to water precipitation in spring‐summer.

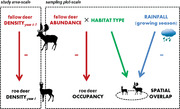

## INTRODUCTION

A shared use of a scarce resource is expected to trigger competition between species through negative effects on growth, fecundity, and/or survivorship of competitors (de Boer & Prins 1990). Interspecific competition is a complex process influencing individual‐ and population‐level patterns such as behavior, physiology, habitat use, local abundance, and distribution, ultimately affecting long‐term population trends (e.g. Putman 1996; Palomares & Caro 1999; Arsenault & Owen‐Smith 2002; Donadio & Busirk 2006; Bao *et al*. 2017; Fattorini *et al*. 2018). Phenology of interspecific interactions is driven by the physical environment, such as habitat quality and weather, as well as by phenotypic traits depending on species life‐history (e.g. Arsenault & Owen‐Smith 2002; Wilmers & Getz 2005; Donadio & Buskirk 2006; Anderwald *et al*. 2016).

Weather is a key determinant of animal population trends because it may elicit both direct and indirect (e.g. resource‐mediated) costs for individuals (Roy *et al*. 2001; Clutton‐Brock & Coulson 2002; Post *et al*. 2009). Interspecific interactions may amplify (Mason *et al*. 2014), buffer (Wilmers & Getz 2005), or add up to (Ferretti *et al*. 2019b) the effects of weather on behavior and ecology of species. By influencing vegetation patterns such as plant growth, viability, and dispersion, weather has the potential to determine resource availability, ultimately driving the potential for competitive interactions between herbivores (Arsenault & Owen‐Smith 2002). However, information on the interplay between interspecific competition and weather is scarce, especially concerning the role of weather in influencing interspecific niche overlap/partitioning.

Among mammalian herbivores, environmental conditions (e.g. habitat quality, food availability, weather) in spring–summer, i.e. during nursing/weaning of offspring, are crucial determinants of population dynamics (e.g. Clutton‐Brock *et al*. 1984; Festa‐Bianchet & Jorgenson 1997; Côté & Festa‐Bianchet 2001; Pettorelli *et al*. 2007). Effects of favorable environmental conditions may be undermined by the presence of competitors at high density, through exploitation of resources and/or interference (Ferretti *et al*. 2011b, 2019b; Mason *et al*. 2014). In this case, a species would be affected by interspecific competition even in good environmental conditions. Alternatively, favorable effects of weather on resource availability could buffer negative effects of interspecific competition (Arsenault & Owen‐Smith 2002). If so, numbers/distribution of a species would be affected by interspecific competition only under harsh environmental conditions. Moreover, the response of species to competition pressure may differ across habitats, as it is expected to be generally stronger in those characterized by a lower availability of suitable resources (Anderwald *et al*. 2016). Evaluating the relative importance of interspecific versus environmental (e.g. habitat, weather) factors in affecting ecology of species would be fundamental to predict population consequences of environmental changes.

In this work, we considered a wild herbivore, the roe deer *Capreolus capreolus*, and studied interactions between its density and occupancy in relation to habitat type, rainfall, and density of its main local competitor (the fallow deer *Dama dama*: Ferretti *et al*. 2011b) in a Mediterranean coastal area, over a 11‐year temporal scale. We also investigated relationships between competitor densities, rainfall, and habitat‐specific spatial overlap between the 2 deer species. The roe deer is well adapted to wood‐field ecotones and relies on highly nutritious vegetation for survival and reproduction (Andersen *et al*. 1998; “concentrate selector,” *sensu* Hofmann 1989). In this ungulate, offspring survival and female reproductive success are strongly influenced by environmental conditions in spring‐summer (Gaillard *et al*. 1992, 1997; Pettorelli *et al*. 2005; McLoughlin *et al*. 2007). In particular, adequate rainfall in spring–summer enhances primary productivity, which is expected to favor body conditions of roe deer females, thus improving their fecundity (Gaillard *et al*. 1992). Conversely, the fallow deer is able to use both nutritious and fibrous vegetation (“intermediate feeder” *sensu* Hofmann 1989) and is not native to our study area, where its last release dates back to the mid‐1960s. Generally, intermediate feeders should be more competitive than concentrate selectors, as they can adapt to a wider dietary spectrum (Hofmann 1989). Indeed, a potential for competition between these 2 deer species has been detected in several study systems, through overlap in diet, habitat, and/or space use (Batcheler 1960; Putman & Sharma 1987; Putman 1996; Focardi *et al*. 2006). In particular, negative effects of fallow deer numbers over roe deer ones have been reported from high latitudes to sub‐arid areas (Putman & Sharma 1987; Focardi *et al*. 2006; Imperio *et al*. 2012; Elofsson *et al*. 2017). Furthermore, fallow deer can displace roe deer from shared feeding grounds through direct aggression; the negative effects of the density of the former have also been shown on small scale distribution and density of the latter, on a short‐term temporal scale (4 years: Ferretti *et al*. 2008, 2011a,b, 2012; Ferretti 2011).

Here, we evaluated (i) population trends of roe deer in relation to those of fallow deer and rainfall variations, (ii) effects of local abundance of fallow deer, rainfall, and habitat on local occupancy of roe deer, (iii) effects of rainfall and local abundance of fallow and roe deer on habitat‐specific spatial overlap between the 2 cervids. We predicted that (i) density of roe deer would be negatively affected by fallow deer density (Putman & Sharma 1987; Focardi *et al*. 2006; Ferretti *et al*. 2011b; Imperio *et al*. 2012) and positively influenced by rainfall in the previous spring–summer (Gaillard *et al*. 1997, 1992); (ii) occupancy of roe deer would be negatively affected by fallow deer local abundance (Ferretti *et al*. 2011b), with no difference across habitats (Ferretti *et al*. 2012). According to optimal foraging theory (MacArthur & Pianka 1966; Schoener 1971), species are expected to narrow their niches when resources are not limiting, which would promote interspecific coexistence through niche partitioning. Conversely, when resources are scarce, species would increase their niche breadth to exploit also sub‐optimal resources. In Mediterranean areas, spring–summer rainfall enhances plant productivity (e.g. Figueroa & Davy 1991; Maselli *et al*. 2014), thus nutritional quality of habitat for ungulates. If so, we predicted that (iii) habitat‐specific spatial overlap, thus potential for competition, would increase with low rainfall/high temperature in spring–summer and, additionally, with habitat‐specific density of fallow deer rather than that of roe deer, due to avoidance of the former by the latter (Ferretti *et al*. 2011b).

## MATERIALS AND METHODS

### Study Area

Our study was conducted in a sector (*c*. 70 km^2^) of the Maremma Regional Park (MRP; Central Italy, 2°39′N, 11°05′E; *c*. 90 km^2^), throughout 11 years (2007–2017). The climate is Mediterranean, with hot‐dry summers and mild‐wet autumns and winters (2007–2017, annual rainfall: 549.4 mm; mean annual temperature: 15.6 °C). Our study area was located south of the Ombrone river, mainly encompassing Uccellina hills (maximum altitude: 417 m a.s.l.). Vegetation is made up by Mediterranean sclerophyllic scrubwood (58%) of 3 main wood types (Mencagli & Stefanini 2008): oakwood, with prevalence of holm oak *Quercus ilex* trees with a canopy height >7 m; scrubland, with prevalence of holm oak, mock privet *Phyllirea* spp., and strawberry tree *Arbutus unedo*, with a height between 2–7 m; garigue, with bushes less than 2 m high (mainly holm oak, rosemary *Rosmarinus officinalis*, juniper *Juniperus* spp., rockrose *Cistus* spp.). Other habitats are pinewood (10%: mainly domestic pine *Pinus pinea*), ecotones between wood and open areas (15%, mainly abandoned olive groves and pastures), set‐aside grassland (4%), and crops (12%, mainly cereals and sunflower).

Wild ungulates included fallow deer, roe deer, and wild boar *Sus scrofa*; large predators included wolves *Canis lupus*. Although there are no data on the potential effects of predation on local ungulate populations, food habit and prey selection studies indicate that fallow deer was the local main prey and roe deer use was relatively low (Manghi & Boitani 2012; Ferretti *et al*. 2019a). Selective culling of fallow deer and wild boar, as well as captures of wild boar with population control purposes, was conducted by Park Wardens and authorized operators (fallow deer culling) throughout the study. Previous work supported no long‐term effects of culling on physiology and distribution of fallow deer (Pecorella *et al*. 2016). In 2011–2016, 18–28% of fallow deer population estimated in summer has been culled each year, leading to the observed reduction in fallow density (see Supporting Information 1, for details).

### Data Collection

We estimated densities of roe deer and fallow deer during 11 years (in 2007 and 2009–2017), in summer (June–early August). We used pellet group counts, a method that has been consistently used since many decades to estimate deer densities in wooded areas with scarce visibility of animals (e.g. Bailey & Putman 1982; Putman 1984; Mayle 1996; Latham *et al*. 1997; Borkowski 2004; Campbell *et al*. 2004; Acevedo *et al*. 2010; Marcon *et al*. 2019), and has been used also in our study area (Fattorini *et al*. 2011; Ferretti *et al*. 2016, 2011b). We used the fecal accumulation rate technique to avoid potential issues related to the estimate of the decay rates of pellet groups, which vary between habitats (Mayle *et al*. 1999; Campbell *et al*. 2004; see Minder 2006, for our study area). We placed a total of 258 circular plots (5 m radius) onto the study area through a stratified sampling design based on major habitat/land cover features. Our sampling strategy is detailed in Supporting Information 2.

Each year, we conducted a first survey to remove all pellet groups from sampling plots. After approximately 35–40 days (according to the local decay rate of deer pellet groups: Massei *et al*. 1998; Minder 2006), we conducted a second survey to count pellet groups in sampling plots (>5 pellets, Mayle *et al*. 1999). We told pellets of fallow apart from those of roe deer according to shape and size: the former defecates cylindrical pellets, usually with a pointed end and slightly concave at the other, whereas the latter makes small, elongated pellets, usually rounded at both ends (Mayle *et al*. 1999). Only one of us (FF) performed pellet group counts throughout the study, to limit subjectivity in pellet identification. For fallow deer, we used a defecation rate of 25 pellet groups/day (in our same study area: Massei & Genov 1998). For roe deer, as local information on defecation rate was lacking, we used the recommended estimated value of 20 pellet groups/day (Mitchell *et al*. 1985; Ratcliffe & Mayle 1992; Mayle *et al*. 1999). As our aim was to assess the relative variation of population densities rather than actual population densities, the use of defecation rate of roe deer from literature is unlikely to affect our conclusions.

Daily rainfall for our study area was recorded at Rispescia station and was provided by Servizio Idrologico Regionale (Regione Toscana). We defined the growing season (5 months) as the period from March to July, i.e. from the beginning of vegetation growth until the count of deer in our study area. Based on rainfall and temperature recorded in this period, we calculated different drought indices to discriminate between years with more and less arid growing seasons (see Supporting Information 3).

### Effects of fallow deer on roe deer density

**Figure 1:**
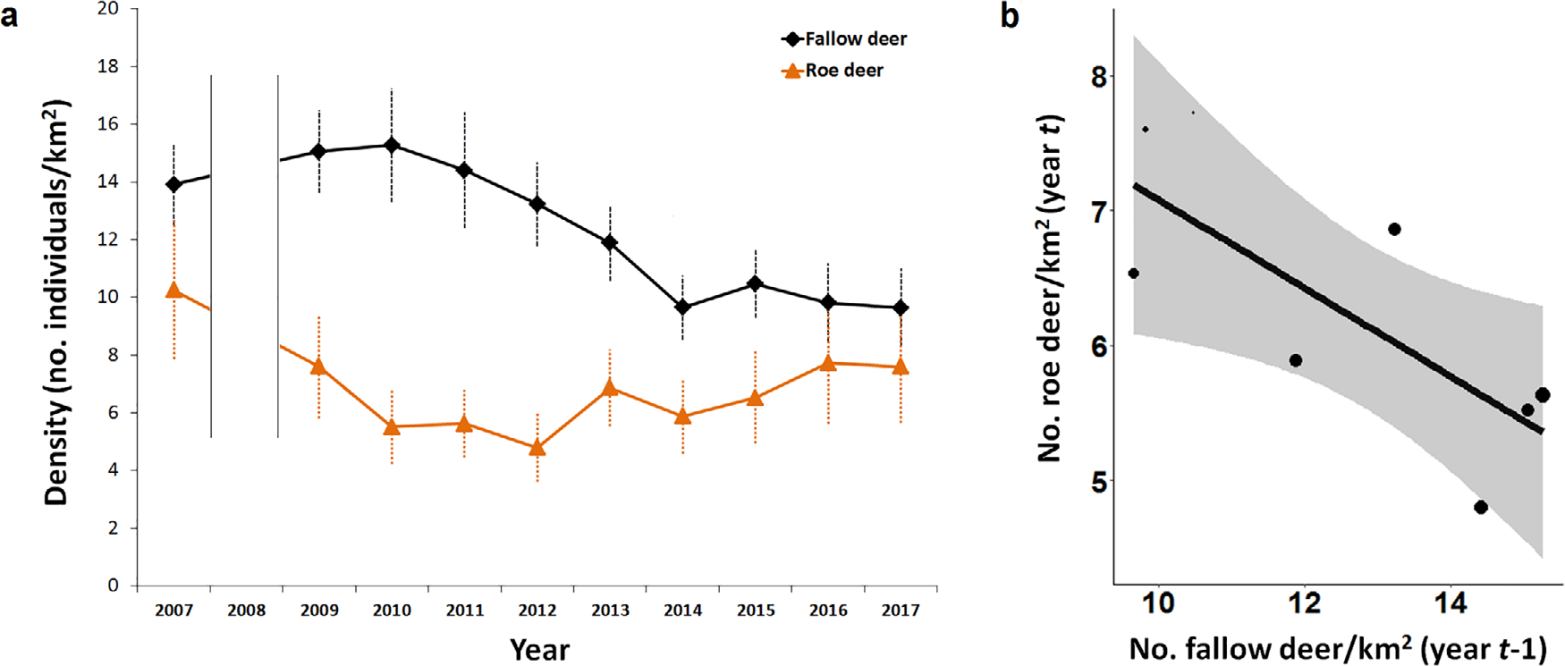
(a) Mean densities of roe and fallow deer estimated at the study area‐scale in 2007–2017 (count in 2008 is missing; orange items: roe deer; black items: fallow deer; error bars: standard error). (b) Predicted annual density of roe deer in relation to density of fallow deer in previous year, at the study area‐scale (lines and bands: predicted values and 95% confidence intervals; dots: observed annual density estimate whose size is weighted by the inverse of precision).

We evaluated interspecific relationships of densities using a weighted linear model (LM; Zuur *et al*. 2009). The response variable was the density of roe deer at the study area‐scale (no. roe deer/km^2^), which was modeled through Gaussian errors (link: identity). We included as predictors the density of fallow deer in both the same and the previous years (continuous variables: no. individuals/km^2^), due to potential delayed effect of competitor density (e.g. Imperio *et al*. 2012; Corlatti *et al*. 2019). We also included the density of roe deer in previous year, to account for density‐dependence in population dynamics of ungulates (e.g. Creel & Creel 2009; Imperio *et al*. 2012; Corlatti *et al*. 2019). In herbivorous mammals, population density is also expected to be influenced by climatic conditions during the previous year. This is especially valid in semi‐arid climates such as in Mediterranean areas, due to potentially negative effects of aridity on vegetation productivity leading to reduced investment in reproduction and offspring survival (Garel *et al*. 2004; Focardi *et al*. 2008; see Gaillard *et al*. 1997; Toïgo *et al*. 2006, for roe deer). In turn, we also set as predictor the cumulative spring–summer rain fallen in our study area in the previous year (continuous, in mm), as a proxy for vegetation productivity. We only considered rainfall because it was collinear with temperature, and we showed that aridity, i.e. drought stress, was mainly related to rainfall in our study years (see Supporting Information 3). Owing to the missing data of densities in 2008, this model concerned 8 years of data (2010–2017). Our density estimates obtained through pellet group count show a relatively high precision (relative standard error in 2007–2017: 10–20%; Fig. 1a; see also Ferretti *et al*. 2011a). However, to account for precision of density estimates, we weighted the annual roe deer density according to the inverse of its confidence interval.

### Effects of fallow deer on roe deer occupancy

We used sampling plot‐specific data obtained by pellet group count surveys to evaluate the effects of fallow deer local abundance on local presence and abundance of roe deer. In particular, we were interested to test whether effects of fallow deer on local occupancy of roe deer were habitat‐specific or occurred in all habitats (Ferretti *et al*. 2011b; but see also Ferretti *et al*. 2012), and how rainfall may shape this pattern. To this end, analyses concerned sampling plots where we detected at least one of the two species (Ferretti *et al*. 2011b). We considered 5 main habitat types occurring in MRP (*cf*. Study Area): oakwood, scrubland, garigue, ecotone, and open areas (where we pooled set‐aside grasslands and crops). We did not consider pinewood because no roe deer pellet groups were found in this habitat type, throughout the study.

Through generalized linear mixed models (GLMMs; Zuur *et al*. 2009), we modeled separately two response variables: (i) presence/absence of roe deer in sampling plots (indexed as presence/absence of pellet groups in the plot); (ii) local abundance of roe deer in sampling plots (given by the no. of pellet groups in the plot). We modeled presence/absence of roe deer in sampling plots through binomial errors (link: logit) and local abundance of roe deer at the sampling plot‐scale through negative binomial errors (link: log), as customary when modeling binary and count variables, respectively. For both response variables, models were conducted at 2 levels. Initially, we considered all data by including as predictors the habitat type (categorical; reference level: ecotones), the local abundance of fallow deer (no. of pellet groups), and their interaction, to test whether the effects of fallow deer local abundance on roe deer occupancy was habitat‐dependent. We also included the year and the sampling plot as crossed random intercepts to account respectively for potential sources of inter‐annual weather variability and for repeated surveys within sampling plots. In a second analysis, we ran separate models for each habitat type to investigate the effect of rainfall regime. We included as predictors the annual spring–summer aridity, as arid or non‐arid years (categorical; reference category: arid years), the local abundance of fallow deer (no. of pellet groups) and their interaction, to assess whether the effects of fallow deer local abundance on roe deer occupancy varied with rain conditions, in each habitat type. Using drought indices (see Supporting Information 3), spring–summer aridity was categorized in 2 levels to improve interpretability of interactive effects. We also included the sampling plot identity as random intercept to account for repeated surveys within sampling plots.

### Effects of rainfall and habitat on spatial overlap

Each year, we considered habitat‐level estimates of (i) spatial overlap between roe deer and fallow deer at the sampling plot scale, (Pianka index: Pianka 1974, see Ferretti *et al*. 2011a, for the same species in our study area), and (ii) the proportion of roe deer space used by fallow deer. We calculated Pianka index in each habitat type and year as follows:

$$P=\sum_{i=1}^{M}o_{\mathrm{iF}}o_{\mathrm{iR}}/\sqrt{\sum_{i=1}^{M}o_{\mathrm{iF}}^{2}\sum_{i=1}^{M}o_{\mathrm{iR}}^{2}}$$

where *n* denotes the total number of plots and *O_iF_* and *O_iR_* denote the proportion of fallow and roe deer pellet groups in the *i*‐th plot, respectively. We calculated the proportion of roe deer space used by fallow deer as *n_FR_/n_R_*, where *n_FR_* is the number of plots including pellet groups of both species and *n_R_* is the number of plots with roe deer pellet groups. These indices provide different estimates of spatial overlap. The former indicates the level of space shared between the 2 species, the latter indicates how much of the roe deer space deer was also used by fallow deer. Both indices can range from 0 (no space shared) to 1 (total overlap).

We evaluated the effects of spring–summer rainfall and habitat type on spatial overlap between the 2 deer species using GLMMs. We only considered rainfall because it was collinear with temperature, and we showed that aridity, i.e. drought stress, was mainly related to rainfall in our study years (see Supporting Information 3). Both indices were modeled through Beta errors (link: logit), as customary with continuous proportions. We included as predictors the habitat type (categorical; reference level: ecotones), the spring–summer rainfall of the same year (continuous, in mm), and their interaction, to test habitat‐specific effects of rainfall in affecting the spatial overlap between the 2 cervids. We also included as covariates the sum of roe deer pellets and the sum of fallow deer pellets in the habitat, to test whether spatial overlap was influenced by the local abundance of the 2 species.

### Multi‐model inference

Resource selection functions are defined as any function describing habitat or resource use that is proportional to the probability of use by an organism (Manly *et al*. 2002). Our design‐based sampling strategy accounted for main habitat/land cover categories in the study area, where sampling plots have been allocated to habitat/land cover categories proportionally to their size (Supporting Information 2, for details). Consequently, our GLMMs fitted with habitat type as predictor may be considered as resource selection functions for probability of presence, abundance, as well as spatial overlap of deer. In each global model set, we inspected the potential collinearity between covariates by calculating the Pearson correlation coefficient. All coefficients were <0.5, suggesting no collinearity between explanatory variables. For each of the above model sets, we performed a model selection to fit all the possible models with different combinations of predictors, as each of them could represent a different *a priori* hypothesis (Burnham & Anderson 2002). The null model was also included in model selection, to allow for an assessment of model performance relative to a fixed baseline (Mac Nally *et al*. 2018). Model selection used Akaike's Information Criterion corrected for small sample sizes (AICc) and considered the “nesting rule” (Burnham & Anderson 2002): models were selected if they had AICc ≤ 2, and if their AICc value was lower than that of any simpler alternative (Burnham & Anderson 2002). Standardized model weight was thus calculated within the subset of selected models. Model selection was performed through R package *MuMIn* (Bartoń 2012).

We estimated parameters (B coefficients and 95% confidence intervals) of the best models using the R packages *stats* (R Core Team 2013), *lme4* (Bates *et al*. 2015), and *glmmTMB* (Brooks *et al*. 2017), and tested whether confidence intervals overlapped 0 to assess effects of predictors (Bolker *et al*. 2009; Grueber *et al*. 2011; Leroux 2019). Best models were validated through visual inspection of residuals (Zuur *et al*. 2009). For the LM, we also checked the normality assumption of residuals (Shapiro–Wilk test: *W* = 0.922, *P* = 0.44).

## RESULTS

### Effects of fallow deer on roe deer density

Throughout our study period, fallow deer density peaked in 2007–2010, decreased by approx. 33% in 2011–2014, and then remained relatively stable; conversely, roe deer numbers decreased by more than 50% in 2007–2013, and then showed a slightly positive trend (Fig. 1a). The best model of roe deer density at the study area‐scale supported the effect of density of fallow deer in previous year, but not effects of roe deer density in previous year, fallow deer density in same year, and spring–summer rainfall in previous year (Table S1, Supporting Information 4). In particular, the higher the density of fallow deer in a given year, the lower the density of roe deer in the following year (Table 1; Fig. 1b).

**Table 1:** Best models of (a) roe deer density at the study area‐scale, (b) probability of roe deer presence (presence of pellet groups per plot) and (c) roe deer abundance (no. pellet groups per plot), (d) Pianka index, and (e) proportion of roe deer space used also by fallow deer

Spatial level	Response variable	Predictor	B	95% CI
Study area‐scale	No. roe deer/km^2^ (year *t*)	Intercept	10.341	6.582; 14.100*
		No. fallow deer/km^2^ (year *t‐1*)	−0.326	−0.615; −0.037*
Sampling plot‐scale	Presence of roe deer pellet groups [Plot ID] var = 2.403 [Year] var = 0.029	Intercept	0.468	−0.470; 1.405
		No. fallow deer pellet groups	−0.351	−0.567; −0.134*
		Habitat type (garigue)	3.676	1.784; 5.568*
		Habitat type (oakwood)	0.181	−0.936; 1.297
		Habitat type (open areas)	1.036	−0.093; 2.165
		Habitat type (scrubland)	0.706	−0.544; 1.957
		Habitat type (garigue) × no. fallow deer pellet groups	−2.758	−4.106; −1.410*
		Habitat type (oakwood) × no. fallow deer pellet groups	−0.365	−0.740; 0.009
		Habitat type (open areas) × no. fallow deer pellet groups	−0.943	−1.412; −0.473*
		Habitat type (scrubland) × no. fallow deer pellet groups	−1.071	−1.577; −0.566*
	No. roe deer pellet groups [Plot ID] var = 0.468 [Year] var = 0.003	Intercept	−0.322	−0.758; 0.113
		No. fallow deer pellet groups	−0.184	−0.291; −0.077*
		Habitat type (garigue)	1.096	0.491; 1.700*
		Habitat type (oakwood)	−0.156	−0.658; 0.346
		Habitat type (open areas)	0.034	−0.467; 0.535
		Habitat type (scrubland)	0.039	−0.531; 0.609
		Habitat type (garigue) × no. fallow deer pellet groups	−0.680	−1.053; −0.308*
		Habitat type (oakwood) × no. fallow deer pellet groups	0.012	−0.147; 0.171
		Habitat type (open areas) × no. fallow deer pellet groups	−0.372	−0.614; −0.130*
		Habitat type (scrubland) × no. fallow deer pellet groups	−0.326	−0.557; −0.095*
	Pianka index	Intercept	−0.814	−1.255; −0.374*
		Spring–summer rainfall (mm)	−0.002	−0.004; −0.001*
		Sum no. fallow deer pellet groups	0.006	−0.001; 0.012
	Roe deer space used by fallow deer	Intercept	2.817	2.086; 3.547*
		Habitat type (garigue)	−3.568	−4.574; −2.562*
		Habitat type (oakwood)	−2.688	−3.637; −1.739*
		Habitat type (open areas)	−3.495	−4.496; −2.495*
		Habitat type (scrubland)	−0.072	−0.981; 0.837

Coefficients (B) and 95% confidence intervals (95% CIs) are shown. For GLMMs, variance (var) of random factors is also reported. The reference category for habitat type is ecotone. Asterisks mark the 95% confidence intervals which do not include 0.

### Effects of fallow deer on roe deer occupancy

**Figure 2:**
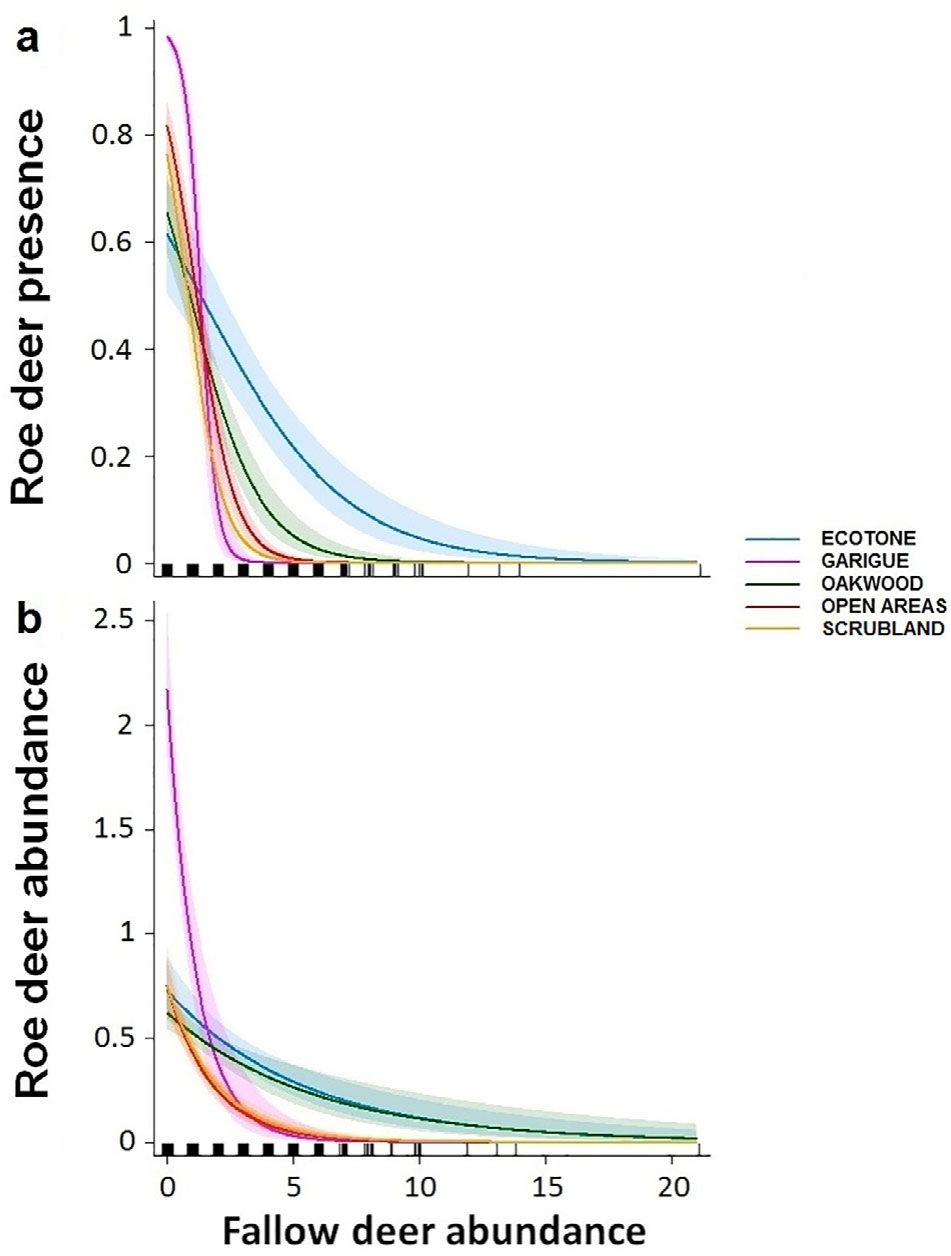
Predicted (a) probability of roe deer presence (presence of pellet groups per plot) and (b) roe deer local abundance (no. pellet groups per plot) in relation to local abundance of fallow deer in sampling plots (no. pellet groups per plot) and habitat type. Lines and bands: predicted values with 95% confidence intervals; *x*‐axis rugs: covariate values.

Both best models of roe deer local presence and abundance supported the interactive effect of habitat type with fallow deer abundance (Table S1, Supporting Information 4). The probability of presence as well as the local abundance of roe deer decreased with increasing local abundance of fallow deer in all habitat types, with a stronger magnitude in garigue, scrubland, and open areas (Table 1; Fig. 2).

Habitat‐specific models further confirmed the above results, as to the effect of fallow deer. Within each habitat type, best models of roe deer local presence and abundance did not support the interactive effect of spring–summer rainfall regime (arid vs. non‐arid) with abundance of fallow deer (Table S2, Supporting Information 4). In all habitat types, both the probability of roe deer presence and local abundance decreased with increasing local abundance of fallow deer (Table S3, Supporting Information 4).

### Effects of rainfall and habitat type on spatial overlap indices

**Figure 3:**
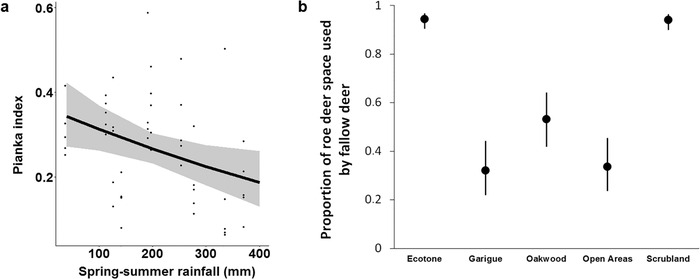
Predicted indices of spatial overlap at the sampling plot‐scale: (a) Pianka index in relation to spring‐summer rainfall (lines and bands: predicted values with 95% confidence intervals; dots: observed values) and (b) proportion of roe deer space used by fallow deer across habitat types (error bars: 95% confidence intervals).

The best model of overall spatial overlap (i.e. Pianka index) between roe and fallow deer supported the negative effect of spring–summer rainfall (i.e. the greater the rainfall, the lower the overlap) and a positive effect of local abundance of fallow deer (i.e. the higher the abundance, the greater the overlap), although the 95% confidence interval of the latter included 0 (Table 1, Supporting Information 4; Fig. 3a).

**Figure 4:**
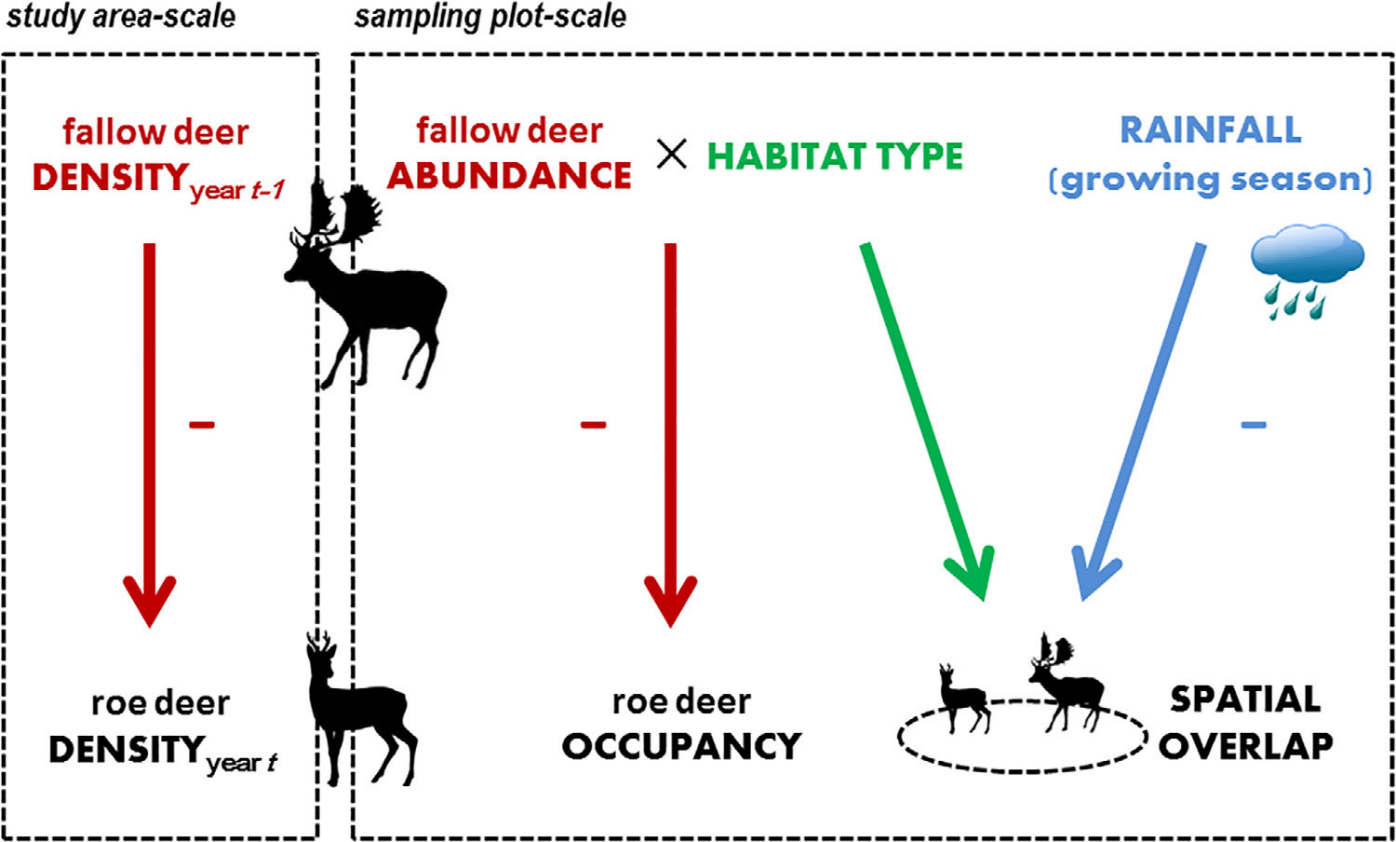
Factors affecting density and local distribution of roe deer, as well as spatial overlap between roe deer and fallow deer, at different spatial scales.

The best model of proportion of roe deer space used also by fallow deer only supported the effect of habitat type (Table S1, Supporting Information 4). This index was greater in ecotones and scrubland than in other habitats (Table 1; Fig. 3b).

## DISCUSSION

There is a growing interest in the relative roles of environmental conditions and interspecific competitive interactions on behavior and ecology of wild herbivores living in temperate ecosystems (e.g. Corlatti *et al*. 2019; Ferretti *et al*. 2019b). Nevertheless, relevant knowledge on herbivore communities inhabiting semi‐arid regions such as Mediterranean ranges is still scarce (but see Imperio *et al*. 2012). Our findings support the role of the fallow deer as a strong competitor to the roe deer (Fig. 4; see also Putman & Sharma 1987; Focardi *et al*. 2006; Ferretti *et al*. 2011b; Imperio *et al*. 2012; Elofsson *et al*. 2017). In particular, we found that (i) the effect of fallow deer density on roe deer numbers was stronger than that of rain conditions, at the study area scale (Imperio *et al*. 2012), (ii) the magnitude of the effect of fallow deer local abundance on roe deer occupancy differed across habitats, and (iii) local interspecific spatial overlap was influenced both by habitat‐specific density of the superior competitor and rainfall, being negatively related to water precipitation in spring–summer.

In Mediterranean regions, where winters are relatively warm, summer is usually the limiting season. Roe deer are considered “income breeders,” i.e. they tend not to store fat reserves but rather invest in reproduction the energy directly obtained from food (Hewison *et al*. 1996; Andersen *et al*. 2000). Thus, dry conditions in spring–summer are expected to limit reproductive success of roe deer females, ultimately affecting population dynamics (Gaillard *et al*. 1997; Pettorelli *et al*. 2005; McLoughlin *et al*. 2007). By contrast, our data did not support an effect of spring–summer rainfall on population density of roe deer, over an 11‐year period, which was rather affected by density of fallow deer in the previous year. In our study area, behavioral interference by fallow deer has been reported mainly in spring–summer, triggering an increase of vigilance behavior in roe deer and negatively affecting its foraging, especially in females (Ferretti *et al*. 2011b). Furthermore, fallow deer density has been shown to negatively affect attendance of pastures as well as small scale distribution of roe deer, strongly supporting a role of interspecific interference in limiting roe deer numbers (Ferretti *et al*. 2011b). Although our 11‐year period may not have been sufficient to capture a considerable spring–summer rainfall variability to test its effect on roe deer density, requiring longer‐term data, we suggest that the effect of competitors may have overwhelmed that of weather (e.g. Mason *et al*. 2014), during our study period.

Beside the delayed effect of competitor density in shaping number of roe deer, our data also suggest effects of competitor on occupancy of roe deer at the same temporal scale. In some “open” areas of our study site, including crops, pastures, and grasslands, behavioral interference between fallow and roe deer was not habitat‐specific (Ferretti *et al*. 2012). Conversely, when all habitat types were considered, we found that the strength of negative effects of fallow deer local abundance on roe deer occupancy was not consistent across habitats. In particular, negative effects of fallow deer densities on local occupancy of roe deer were the smallest in ecotonal habitats and oakwood, whereas the strongest effect was in scrubland, garigue, and open habitats, including cultivated fields and set‐asides. Results support an interactive effect of local abundance of fallow deer and habitat on local distribution of roe deer, with no effect of the growing season aridity. Ecotones are usually optimal habitats for deer species (e.g. McLoughlin *et al*. 2007; Miyashita *et al*. 2008). In our study area, they include olive groves and bushes interspersed with meadows and pastures, providing deer with food and cover, partially buffering the negative effects of fallow deer density on roe deer occupancy. Likewise, oakwood can provide deer with both cover and food (e.g. items linked to forest renovation such as woody shoots and seedlings in coppice stands, substantially present in the diet of roe deer: Minder 2012, in our study area). Conversely, food offer is expected to be low in the Mediterranean scrubwood and especially in the garigue (Minder 2006). Open habitats also include set‐asides where fallow deer reach great local densities (more than 30–40 individuals/km^2^, in summer: Ferretti *et al*. 2011a), forming groups including up to several tens of individuals (Pecorella *et al*. 2019), whereas roe deer summer groups are much smaller, being usually lower than 3–4 individuals (Fattorini & Ferretti 2019). Open habitats could be also “risky” areas, especially at night, providing no cover from predators. Hence, our data suggest that the use of scrubland, garigue, and open areas by roe deer is further reduced by competition, with a moderate density of fallow deer being sufficient to limit roe deer distribution.

Niche partitioning can occur by spatial, temporal, or dietary differentiation. A substantial dietary overlap has been reported between roe deer and fallow deer in our study area (>80%: Manganelli 2012). The dietary niche of fallow deer can include plants preferred by roe deer, in agreement with feeding adaptations of these 2 ruminants, with the latter being a “concentrate selector” and the former an “intermediate feeder” (*sensu* Hofmann 1989). Food availability peaks in spring months (Chines *et al*. 1997; Minder 2006), whereas environmental conditions are more limiting in summer (Massei *et al*. 1997). A substantial browsing pressure has been reported in wooded habitats (approximately 30–80% browsed shoots, Melini *et al*. 2019), and browsing may tend to become increasingly important after spring, i.e. after the seasonal peak of availability of fresh pasture (Chines *et al*. 1997; Minder 2006). In turn, local pressure by fallow deer on natural vegetation could negatively affect food availability for roe deer, reducing habitat quality to it (Putman 1996; Focardi *et al*. 2006).

In another Mediterranean area, fallow deer density has been shown to correlate positively to roe deer home range size, and negatively with roe deer body weight, supporting a negative role of the former on habitat quality for the latter (Focardi *et al*. 2006). These authors concluded that competition by fallow deer determined a sharp reduction of roe deer numbers (by 80%, over 2 years; Focardi *et al*. 2006). The potential of fallow deer to reduce habitat quality for roe deer has been observed also in a forested area in the United Kingdom (Putman & Sharma 1987). Thus, resource exploitation and behavioral interference are unlikely to be mutually exclusive. Rather, they may be two complementary processes of competition, with interference occurring mainly in open habitats (Ferretti *et al*. 2011b, 2012) and resource exploitation limiting food supply to roe deer in forested habitats.

Information on the role of weather variations on niche relationships, thus on potential for competition, is scarce for wild herbivores. Resource abundance should promote interspecific niche partitioning, i.e. coexistence, whereas resource scarcity may stimulate resource overlap through increased searching behavior to meet energetic demands (MacArthur & Pianka 1966; Schoener 1971). In strongly seasonal areas such as Mediterranean ones, summer drought stress is a crucial limiting factor for plant growth due to physiological mechanisms (Pereira & Chaves 1995). Thus, spring–summer rainfall should be expected to increase habitat quality, leading to better foraging conditions for herbivores (e.g. Toïgo *et al*. 2006; Ferretti *et al*. 2019b). Our results are consistent with better environmental conditions, i.e. higher rain fallen in spring‐summer, favoring spatial partitioning between competitors. In fact, we observed that a greater overlap between deer species was related to low rainfall levels, regardless of habitat type and fallow and roe deer local abundance. If so, overlap would increase in more limiting conditions, further supporting competition between these 2 cervids (de Boer & Prins 1990; Putman 1996). Conversely, the proportion of roe deer space used by the superior competitor was not related to rainfall, but peaked in ecotones and scrubland, further confirming the importance of habitat type in shaping interactions between competitors. Whereas we were able to test indirect effects of rainfall levels/aridity, i.e. the weather experienced by deer during the growing season, daily/hourly data of deer distribution would be necessary to investigate direct effects of temperature. Future studies should assess potential direct effects of temperature on space use/activity of roe deer, clarifying its potential interplay with fallow deer presence/abundance in shifting either spatial or thermal niche of roe deer.

Over evolutionary times, species should be expected to limit competitive interactions through niche partitioning (Gause 1934; Pianka 1974; Schoener 1974). Human‐driven alteration of ecological communities such as introductions of alien taxa is a major primer of interspecific competition, if introduced species have ecological requirements similar to those of indigenous ones (e.g. Gurnell *et al*. 2004; Mori *et al*. 2019; see Ferretti & Lovari 2014, for deer). Fallow deer has been frequently reported as intolerant to other ungulate species, at feeding contexts (e.g. Bartoš *et al*. 1996, 2002; McGhee & Baccus 2006). Ferretti *et al*. (2011b) suggested that fallow deer aggressively defend crucial resources from competitors as an adaptive strategy triggered by recent evolution in semi‐arid habitats (Kurtén 1968). As intermediate feeder, the fallow deer can adapt to a relatively wide food spectrum, enhancing its competitiveness. If so, the spread of this ecologically flexible, large, and gregarious ungulate species would lead to a great potential for competition with native ungulates (Ferretti & Mori 2019, for a review).

There is a growing concern about the potential negative effects of climatic changes on behavior and ecology of animal populations (Root *et al*. 2003; Parmesan 2006, for reviews). Unlike the global trend of temperature increase (Steffen *et al*. 2018), predictions of world rainfall patterns for the next century highlight a higher geographical variability across continents (e.g. Gonzalez *et al*. 2018). However, in the Mediterranean basin, different forecasts have predicted an univocal tendency (Goubanova & Li 2007; Cramer *et al*. 2018), with summer rainfall decreasing up to 9% with each °C increase (Lionello & Scarascia 2018), and dry season lengthening from 1 to 3 weeks owing to 4 °C warming, during the 21th century (Giannakopoulos *et al*. 2009). Although we did not detect significant relationships between roe deer density and rainfall over an 11‐year temporal scale, or an effect of rainfall on roe deer occupancy, we found that a decrease in spring–summer rainfall increased the spatial overlap between roe deer and its competitor. The potential for growing temperatures to negatively affect roe deer numbers has been emphasized (Plard *et al*. 2014; Gaillard *et al*. 2013). In particular, early onset of spring can trigger a mismatch between the peak of resource availability and that of births, with effects on reproductive success of roe deer females (Plard *et al*. 2014). Although individual‐based data on fertility, reproductive success, and mortality are still required to unravel mechanisms underlying the observed pattern of roe deer numbers, our work suggests that lower rainfall elicits the potential for competition with a generalist herbivore, posing additive threats to roe deer populations. Especially in semi‐arid regions such as Mediterranean areas, relationships between weather, population dynamics, and competition of wild herbivores should be further investigated.

## CONFLICT OF INTEREST

The authors declare they have no conflict of interests.

## Supporting information

**Supporting Information 1** Variation of fallow deer density in relation to selective culling

**Supporting Information 2** Sampling strategy

**Supporting Information 3** Climatic data analysis criteria and assessment of drought indices

**Supporting Information 4** Results of model selection analyses
