# Supplementary material for: Competitor densities, habitat, and weather: effects on interspecific interactions between wild deer species
Source: Integr Zool. 2020 Aug 19;16(5):670–84. doi: 10.1111/1749-4877.12470 (PMC8451872; doi:10.1111/1749-4877.12470)

Supporting Information 1

Francesco Ferretti & Niccolò Fattorini

Research Unit of Behavioural Ecology, Ethology and Wildlife Management,
Department of Life Sciences, University of Siena,
Siena, Italy

Competitor densities, habitat, and weather: effects on interspecific interactions between wild deer species

The annual fallow deer density estimated in our study area (n. fallow deer/km^2) showed a moderate, negative correlation to number of fallow deer culled in the previous 12 months (r=-0.36; Fig. S1a), whereas it showed a strong, negative correlation to number of fallow deer culled in the previous 24 months (r=-0.61; Fig. S1b). Thus, selective control at the two-year time-lag was effective to reduce fallow deer density in our study area, explaining its decrease between 2007-2017 (*cf*. Figure 1a, main text).

Figure S1. Annual density of fallow deer in relation to number of fallow deer harvested (a) in the previous 12 months and (b) in the previous 24 months, at the study area-scale. Linear regression lines were added to emphasize trends.

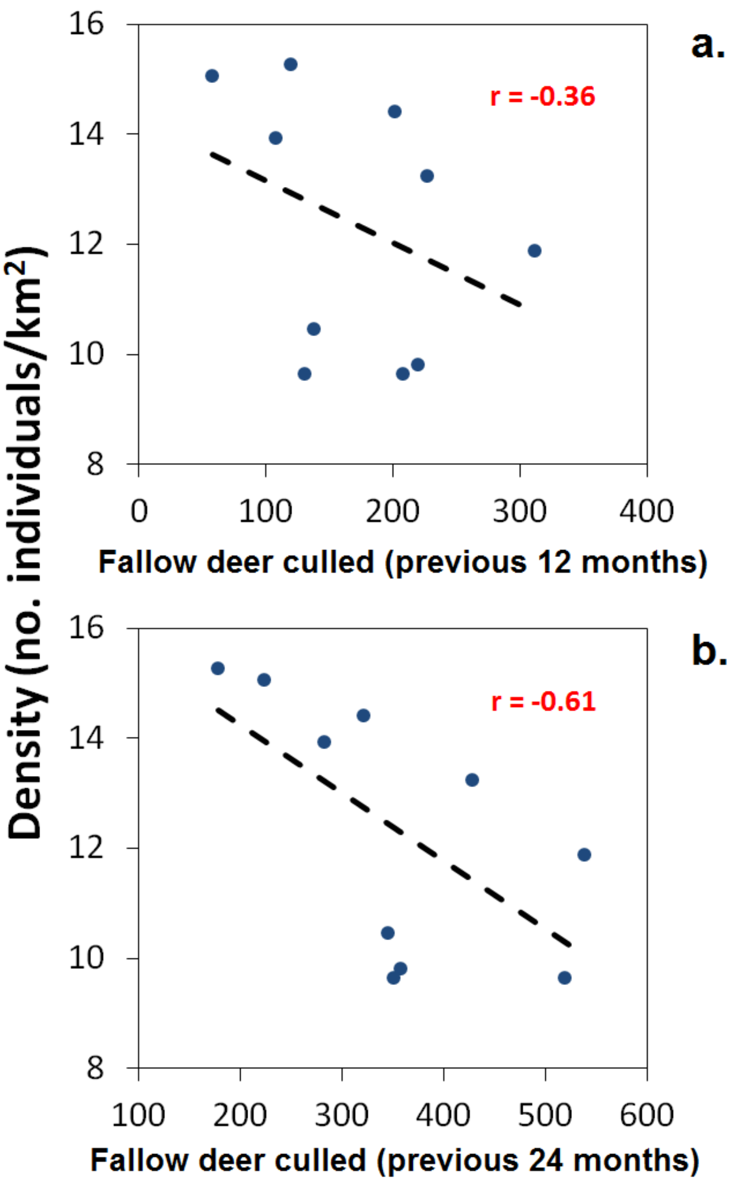

Supplement: Supplementary file 1 — Supporting Information 1 Variation of fallow deer density in relation to selective culling [file INZ2-16-670-s002.pdf]
