# Supplementary material for: Competitor densities, habitat, and weather: effects on interspecific interactions between wild deer species
Source: Integr Zool. 2020 Aug 19;16(5):670–84. doi: 10.1111/1749-4877.12470 (PMC8451872; doi:10.1111/1749-4877.12470)
Supplement: Supplementary file 2 — Supporting Information 2 Sampling strategy [file INZ2-16-670-s001.pdf]

Supporting Information 2

Francesco Ferretti & Niccolò Fattorini

Research Unit of Behavioural Ecology, Ethology and Wildlife Management,
Department of Life Sciences, University of Siena,
Siena, Italy

Competitor densities, habitat, and weather: effects on interspecific interactions between wild deer species

Sampling strategy

We placed a total of 258 circular plots (5 m radius) onto the study area through a stratified sampling design (Gregoire & Valentine 2008). Strata were determined according to the main habitat categories: Mediterranean scrubwood; pinewood; abandoned olive groves and pastures; set-aside grassland; cultivated fields (Fattorini et al. 2011; Ferretti et al. 2011a). We also considered local features as well as differences in local deer densities, assessed through preliminary pellet group counts (Fattorini et al. 2011) and divided Mediterranean scrubwood and abandoned olive groves and pastures in two strata (North and South), while crops were divided in 5 strata (Fattorini et al. 2011; Ferretti et al. 2011). A two-stage strategy was adopted in larger strata (north/south Mediterranean scrubwood, pinewood and two strata of cultivated fields), which were initially partitioned into spatial units of different sizes. A sample of units was selected through a probabilistic sequential (draw-by-draw) sampling scheme. Selection probabilities of units were proportional to unit size, to handle the presence of units with different sizes (Skalski 1994). We did not select contiguous units, which were likely to be more similar than separate ones, thus giving poor contribution to sample information (Fattorini 2006; Grafström & Tillé 2013). Subsequently, a grid of polygons (e.g., quadrats) of equal size was overlain to each unit, and a plot was randomly placed within each of them (tessellation stratified sampling, see Fattorini et al. 2011). This scheme provides an even distribution of plots within units, in respect to that obtained through a completely random allocation. In smaller strata, plots were placed directly onto the stratum through the tessellation stratified sampling. Plots were allocated to strata proportionally to their size. Methodological details and theoretical justifications are given in Fattorini et al. (2011), where an unbiased estimator of feces abundance and a conservative estimator of its standard error are provided.

References

Fattorini L (2006). Applying the Horvitz-Thompson criterion in complex designs: a computer-intensive perspective for estimating inclusion probabilities. *Biometrika* **93**, 269-278.

Fattorini L, Ferretti F, Pisani C, Sforzi A (2011). Two-stage estimation of ungulate abundance in Mediterranean areas using pellet group count. *Environmental and Ecological Statistics* **18**, 291-314.

Ferretti F, Bertoldi G, Sforzi A, & Fattorini L (2011). Roe and fallow deer: are they compatible neighbours? *European Journal of Wildlife Research* **57**, 775-783.

Grafström A, Tillé Y (2013). Doubly balanced spatial sampling with spreading and restitution of auxiliary totals. *Environmetrics* **24**, 120-131.

Skalski JR (1994). Estimating wildlife population based on incomplete area surveys. *Wildlife Society Bulletin* **22**, 192-203.
