# Supplementary material for: Competitor densities, habitat, and weather: effects on interspecific interactions between wild deer species
Source: Integr Zool. 2020 Aug 19;16(5):670–84. doi: 10.1111/1749-4877.12470 (PMC8451872; doi:10.1111/1749-4877.12470)
Supplement: Supplementary file 3 — Supporting Information 3 Climatic data analysis criteria and assessment of drought indices [file INZ2-16-670-s004.pdf]

Supporting Information 3

Francesco Ferretti & Niccolò Fattorini

Research Unit of Behavioural Ecology, Ethology and Wildlife Management,
Department of Life Sciences, University of Siena,
Siena, Italy

Competitor densities, habitat, and weather: effects on interspecific interactions between wild deer species

Climatic data analysis criteria and assessment of drought indices

We defined the growing season (5 months) as the period from March to July, i.e. from the beginning of vegetation growth until the count of deer in our study area. We used different drought indices to discriminate between years with more and less arid growing seasons. In particular, for each growing season, we calculated: (*i*) the mean rainfall–temperature index (a monthly index equal to the ratio between monthly rainfall and mean monthly temperature, whereby values <2 indicate aridity; Gaussen 1954); (*ii*) the compensated drought index IOS_3 (the summation of monthly rainfall divided by the summation of mean monthly temperatures, whereby values <2 indicate aridity; Rivas-Martinez et al. 1999). The former characterizes the mean number of arid months. The latter also account for possible compensation of rainfall in the previous months. Both indices have been used in studies on wild ungulates (e.g. Gaillard et al. 1997; Garel et al. 2004; Toïgo et al. 2006; Focardi et al. 2008; Richard et al. 2010).

In our study years (2007 and 2009-2017), both indices agreed in identifying 4 arid and 6 non-arid years (Figure S1). This grouping (arid vs non-arid years) was further confirmed by a discriminant analysis conducted in relation to total rainfall R and mean temperature T (Group=53.5×R-0.3×T; variance explained: 100%; PERMANOVA test on original variables: F=16.18, p<0.01, 99999 permutations; Monte Carlo permutation t test on discriminant scores, t=-4.8, p<0.01, 99999 permutations). Accordingly, both temperature and rainfall differed significantly between arid and non-arid years (Monte Carlo permutation t test with 99999 permutations; mean temperature: t=2.65, p=0.03; total rainfall: t=-4.02, p=0.008; Figure S2). However, total rainfall correlated more to discriminant scores (r=0.95) than temperature did (r=-0.79), meaning that the former has the main effect in explaining difference between arid vs non arid years. Given the collinearity of temperature and rainfall (r=-0.6; Figure S3a), we thus decided to use the rainfall as a proxy of aridity when performing models (cf. main text).

We could not assess direct effect of temperature, because daily/hourly data on occupancy of deer would be required and modelled in relation to daily/hourly temperatures. Despite mean temperature during months of data collection (June-July) might be a proxy for the direct effect of temperature on deer occupancy at a coarse temporal-scale, the mean temperature during data collection was also strongly collinear to the rain fallen during the growing season (r=-0.7; Figure S3b), meaning that in arid years deer also experienced the hottest summer temperatures. Thus, it would not be possible to include both aridity and temperature of data collection period in the same model to disentangle relevant effects.

Figure S1. Drought indices of growing season (black: mean rainfall–temperature index; blue: compensated drought index IOS_3) over the study years. The red line indicates the threshold value under which the growing season can be considered as arid (see above).

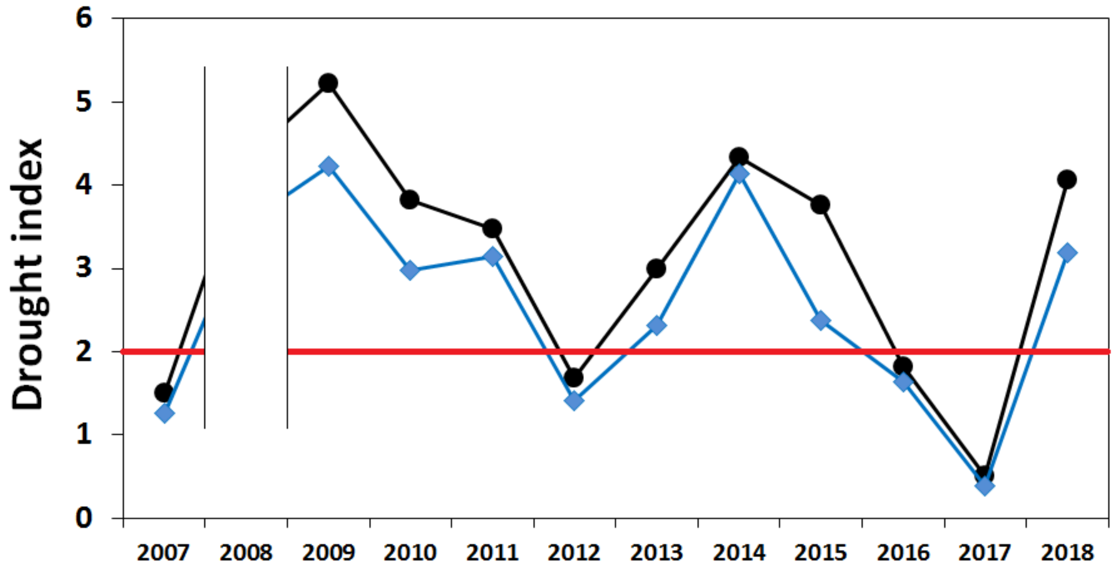

Figure S2. Boxplots (median, quartiles and range) and bar charts (mean and standard error) showing differences in mean temperature (a) and total rainfall (b) of the growing season between arid and non-arid years. Difference is significant for both temperature and rainfall (see above).

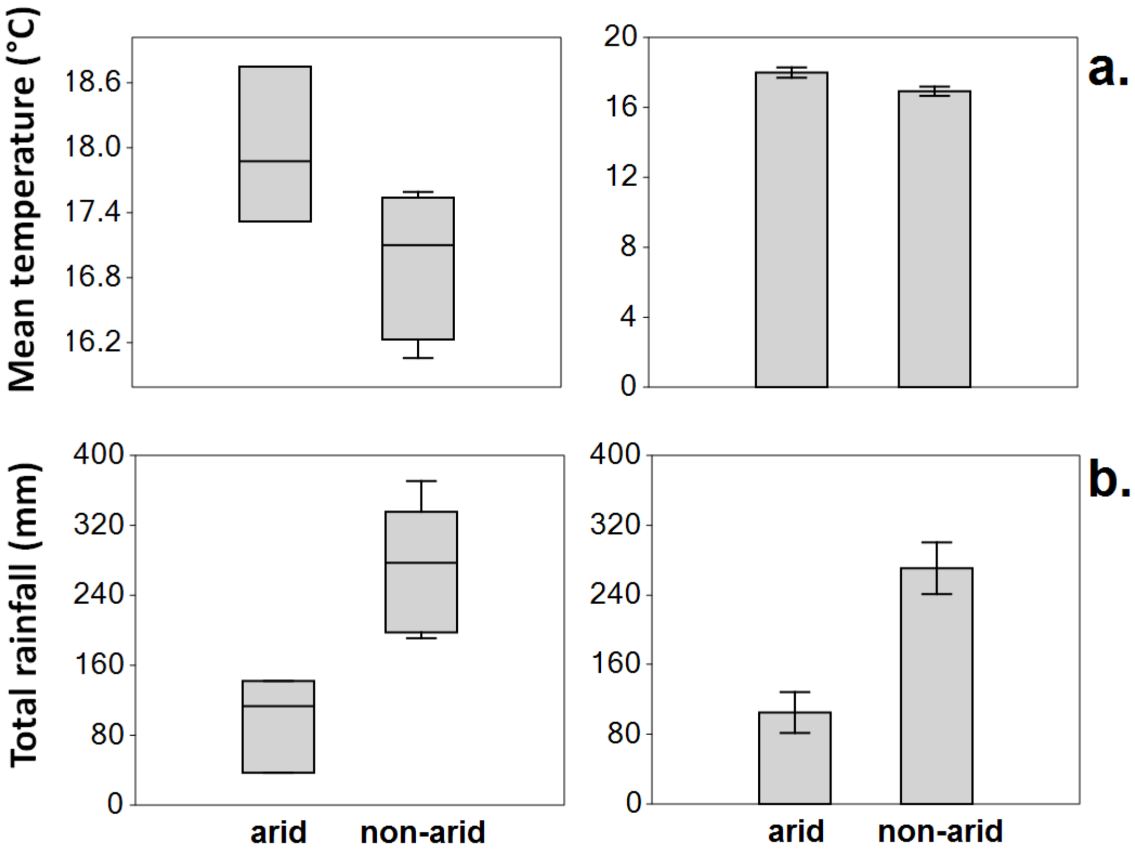

Figure S3. Relationship between (a) mean temperature in the growing season and total rainfall in thegrowing season, and (b) mean temperature during data collection and total rainfall in the growing season, over 10 years (2007 and 2009-2017). Growing season (GS): March-July; data collection (DC): June-July. Red dots: arid years; blue dots: non-arid years. Linear regression lines were added to emphasize trends.

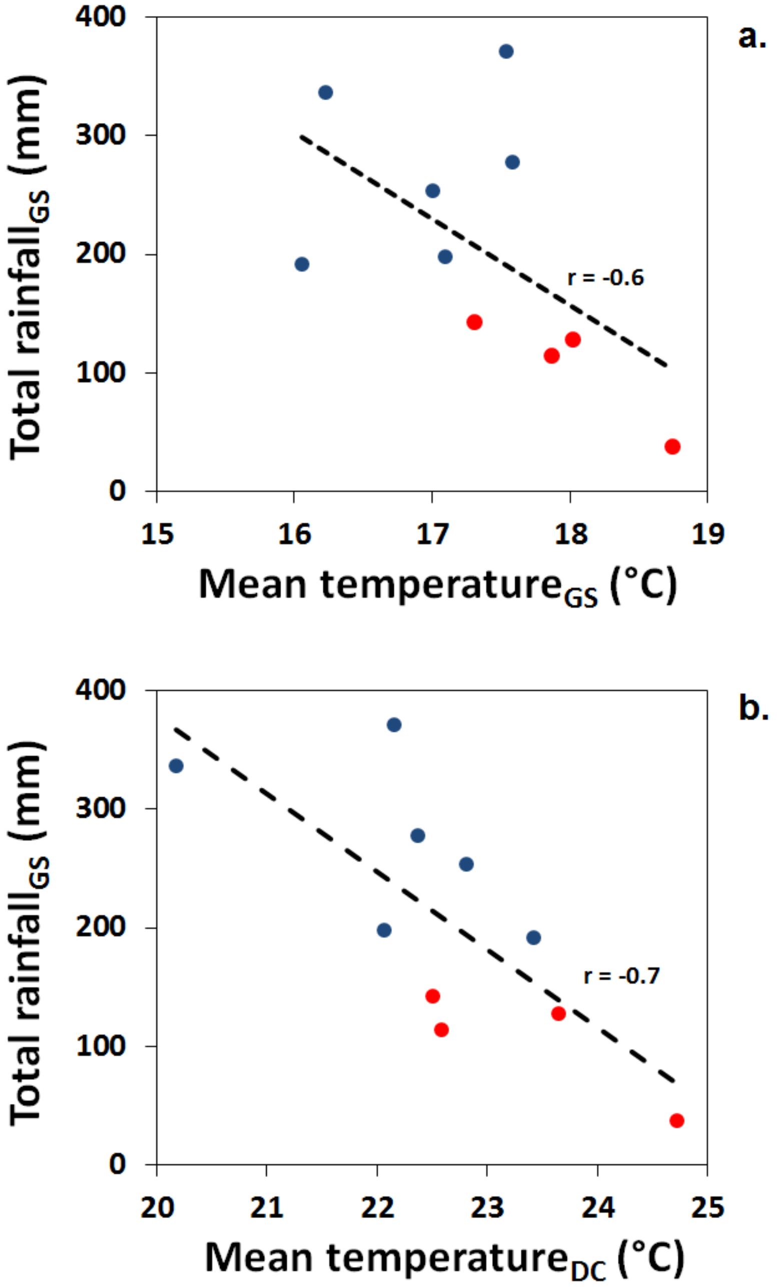

References

Gaillard JM, Boutin JM, Delorme D, Van Laere G, Duncan P, Lebreton JD (1997). Early survival in roe deer: causes and consequences of cohort variation in two contrasted populations. *Oecologia* **112**, 502-513.

Garel M, Loison A, Gaillard JM, Cugnasse JM, Maillard D (2004). The effects of a severe drought on mouflon lamb survival. *Proceedings of the Royal Society of London. Series B: Biological Sciences* **271**, S471-S473.

Focardi S, Gaillard JM, Ronchi F, Rossi S (2008). Survival of wild boars in a variable environment: unexpected life-history variation in an unusual ungulate. *Journal of Mammalogy*, **89**, 1113-1123.

Gaussen H (1954). *Théorie et classification des climats et microclimats*. VIII° Congrès international de botanique. Paris.

Richard E, Gaillard JM, Saïd S, Hamann JL, Klein F (2010). High red deer density depresses body mass of roe deer fawns. *Oecologia* **163**, 91-97.

Rivas-Martinez SD, Sanchez-Mata, Costa M (1999). North American boreal and western temperate forest vegetation (syntaxonomical synopsis of the potential natural plant communities of North America, II). *Itinera Geobotanica* **12**, 5-316.

Toïgo C, Gaillard JM, Van Laere G, Hewison M, Morellet N (2006). How does environmental variation influence body mass, body size, and body condition? Roe deer as a case study. *Ecography* **29**, 301-308.
