# Supplementary material for: Competitor densities, habitat, and weather: effects on interspecific interactions between wild deer species
Source: Integr Zool. 2020 Aug 19;16(5):670–84. doi: 10.1111/1749-4877.12470 (PMC8451872; doi:10.1111/1749-4877.12470)
Supplement: Supplementary file 4 — Supporting Information 4 Results of model selection analyses [file INZ2-16-670-s003.pdf]

Supporting Information 4

Francesco Ferretti & Niccolò Fattorini

Research Unit of Behavioural Ecology, Ethology and Wildlife Management,
Department of Life Sciences, University of Siena,
Siena, Italy

Competitor densities, habitat, and weather: effects on interspecific interactions between wild deer species

Table S1. Result of model selection while accounted for nesting: selected models within ΔAICc<2, each with k, AICc value, ΔAICc and standardized weight. Random factors are shown in square brackets.

Spatial level	Response variable	Selected models	k	AICc	ΔAICc	Weight
Study area-scale	No. roe deer/km^2 (year *t*)	No. fallow deer/km^2 (year *t*-1)	3	27.1	0	0.618
		null model	2	28.1	1	0.382
Sampling plot-scale	Presence roe deer pellet groups	Habitat type × No. fallow deer pellet groups + [Plot ID] + [Year]	12	1323.4	0	1
	No. roe deer pellet groups	Habitat type × No. fallow deer pellet groups + [Plot ID] + [Year]	13	2591.3	0	1
	Pianka index	Spring-summer rainfall (mm) + Sum no. fallow deer pellet groups	4	-72.8	0	0.622
		Spring-summer rainfall (mm)	3	-71.8	1	0.378
	Roe deer space used by fallow deer	Habitat type	6	-168.2	0	1

Table S2. Result of model selection while accounted for nesting: selected models within ΔAICc<2, each with k, AICc value, ΔAICc and standardized weight. Random factors are shown in square brackets.

Habitat type	Response variable	Selected models	k	AICc	ΔAICc	Weight
Ecotone	Presence roe deer pellet groups	Spring-summer aridity + No. fallow deer pellet groups + [Plot ID]	4	251.6	0	0.597
		No. fallow deer pellet groups + [Plot ID]	3	251.4	0.79	0.403
	No. roe deer pellet groups	No. fallow deer pellet groups + [Plot ID]	4	417	0	1
Garigue	Presence roe deer pellet groups	No. fallow deer pellet groups + [Plot ID]	3	74.3	0	1
	No. roe deer pellet groups	No. fallow deer pellet groups + [Plot ID]	4	323.7	0	1
Oakwood	Presence roe deer pellet groups	Spring-summer aridity + No. fallow deer pellet groups + [Plot ID]	4	438.8	0	1
	No. roe deer pellet groups	No. fallow deer pellet groups + [Plot ID]	4	844.2	0	1
Open Areas	Presence roe deer pellet groups	Spring-summer aridity + No. fallow deer pellet groups + [Plot ID]	4	285	0	0.504
		No. fallow deer pellet groups + [Plot ID]	3	285	0.04	0.496
	No. roe deer pellet groups	No. fallow deer pellet groups + [Plot ID]	4	521.8	0	1
Scrubland	Presence roe deer pellet groups	No. fallow deer pellet groups + [Plot ID]	3	273.7	0	1
	No. roe deer pellet groups	No. fallow deer pellet groups + [Plot ID]	4	466.6	0	1

Table S3. Best models of probability of roe deer presence (presence of pellet groups) and roe deer abundance (no. pellet groups) in sampling plots, for each habitat type, estimated through GLMMs. Coefficients (B) and 95% confidence intervals (95% CIs) are shown. Variance (var) of random factors is also shown. Asterisks mark the 95% confidence intervals which do not include 0.

Habitat type	Response variable	Predictor	B	95% CI
Ecotone	Presence roe deer pellet groups [Plot ID] var = 1.32	Intercept	0.338	-0.501; 1.178
		Spring-summer aridity (arid)	0.579	-0.096; 1.254
		No. fallow deer pellet groups	-0.369	-0.574; -0.164*
	No. roe deer pellet groups [Plot ID] var = 0.64	Intercept	-0.403	-0.907; 0.101
		No. fallow deer pellet groups	-0.171	-0.280; -0.061*
Garigue	Presence roe deer pellet groups [Plot ID] var = 3.73	Intercept	4.489	2.210; 6.768*
		No. fallow deer pellet groups	-3.320	-4.905; -1.734*
	No. roe deer pellet groups [Plot ID] var = 0.04	Intercept	0.873	0.659; 1.087*
		No. fallow deer pellet groups	-0.926	-1.251; -0.602*
Oakwood	Presence roe deer pellet groups [Plot ID] var = 2.69	Intercept	0.378	-0.303; 1.058
		Spring-summer aridity (arid)	0.610	0.060; 1.160*
		No. fallow deer pellet groups	-0.703	-1.006; -0.399*
	No. roe deer pellet groups [Plot ID] var = 0.87	Intercept	-0.616	-0.965; -0.267*
		No. fallow deer pellet groups	-0.159	-0.286; -0.033*
Open Areas	Presence roe deer pellet groups [Plot ID] var = 3.85	Intercept	1.349	0.543; 2.154*
		Spring-summer aridity (arid)	0.552	-0.203; 1.306
		No. fallow deer pellet groups	-1.328	-1.785; -0.871*
	No. roe deer pellet groups [Plot ID] var < 0.01	Intercept	-0.012	-0.250; 0.227
		No. fallow deer pellet groups	-0.630	-0.835; -0.426*
Scrubland	Presence roe deer pellet groups [Plot ID] var = 1.62	Intercept	1.133	0.377; 1.890*
		No. fallow deer pellet groups	-1.355	-1.798; -0.911*
	No. roe deer pellet groups [Plot ID] var = 0.58	Intercept	-0.289	-0.720; 0.142
		No. fallow deer pellet groups	-0.540	-0.759; -0.321*
